# Supplementary material for: Pervasive chromosomal instability drives the karyotypic evolution of hypodiploid tumours
Source: Genome Med. 2026 May 22;18:70. doi: 10.1186/s13073-026-01632-y (PMC13195901; doi:10.1186/s13073-026-01632-y)
Supplement: Supplementary file 1 — Additional File 1: Supplementary Figures 1-8 [file 13073_2026_1632_MOESM1_ESM.pdf]

Pervasive Chromosomal Instability Drives the Karyotypic Evolution of Hypodiploid Tumours: Supplementary Figures

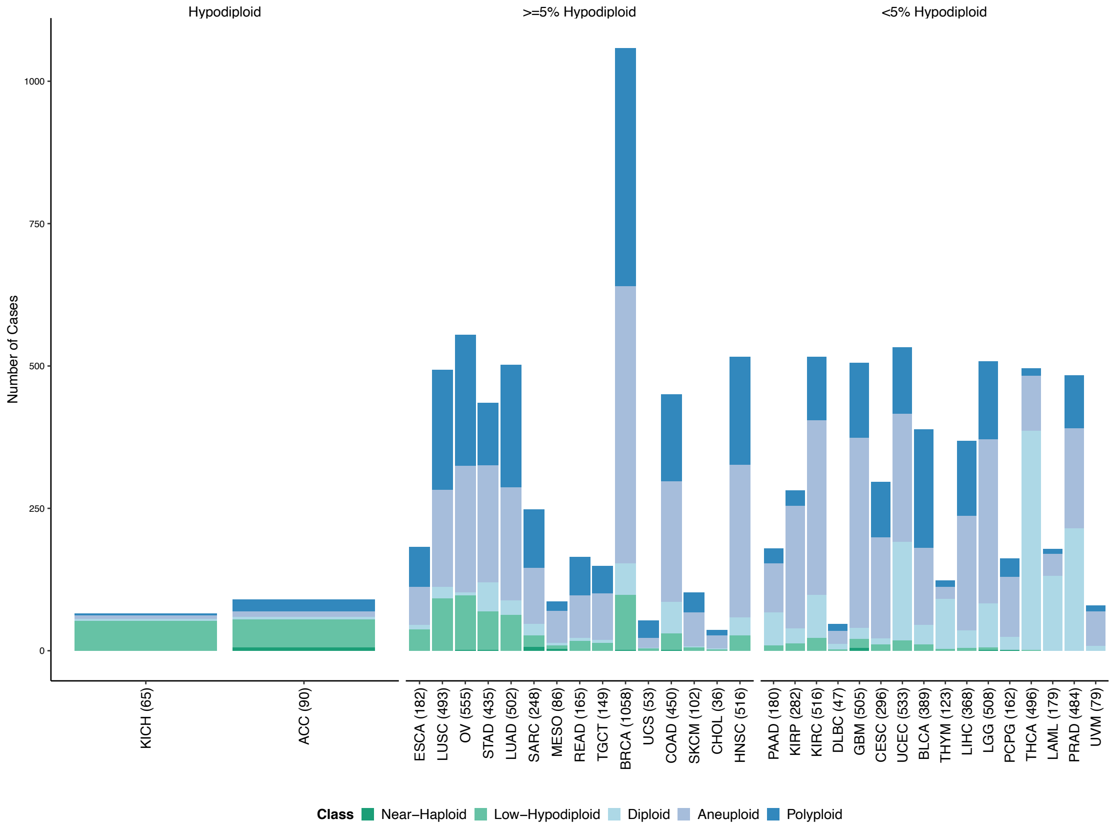

Fig. S1: Related to Fig. 1. Number of cases of each ploidy class per TCGA cohort. Number of cases of each TCGA cancer type that are near-haploid ($<$ 28 autosomes), low-hypodiploid $\leq$ 38 autosomes), diploid (all autosomes disomic), polyploid (positive WGD call and no hypodiploid history), and aneuploid (all other cases). Current and former hypodiploids are both counted as hypodiploid. Tumour abbreviations are reported as per https://gdc.cancer.gov/resources-tcga-users/tcga-code-tables/tcga-study-abbreviations

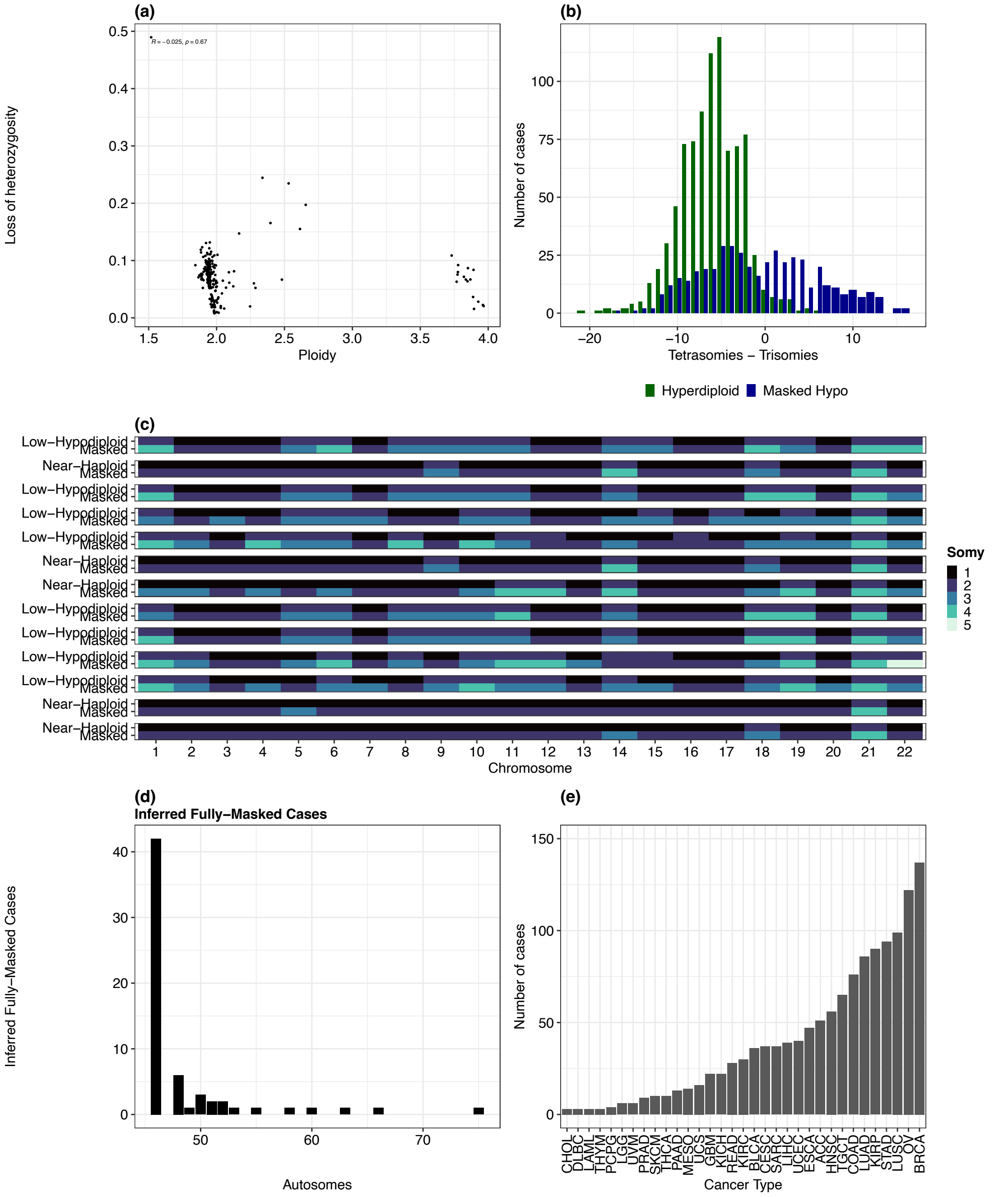

Fig. S2: Related to Fig. 2. Identification of masked hypodiploid ALL based on allele-nonspecific data. A, Distribution of ploidy and loss of heterozygosity among 293 cases from the TARGET-ALL-P2 project. Hypodiploid cases with multiple WGD events would appear in the top right. **B,** Distribution of MH score (# tetrasomies - # trisomies, excluding the sex chromosomes) in the TCGA cases. Masked hypodiploid cases include any near-haploid or low-hypodiploid TCGA case with a positive WGD call; hyperdiploid cases were defined based on the absence of a hypodiploid history and an autosome count between 49 and 65 (adjusted from 51-67 chromosomes in Woodward et al.). See (E) for cancer type distribution. **C,** Visualisation of chromosome counts in false negative cases. Each facet represents a case with two clones; colour indicates chromosome copy number (somy). **D,** Autosome counts of inferred fully-masked hypodiploids in ALL from the Mitelman database. **E,** Distribution of TCGA cohort sample sizes analysed for (B).

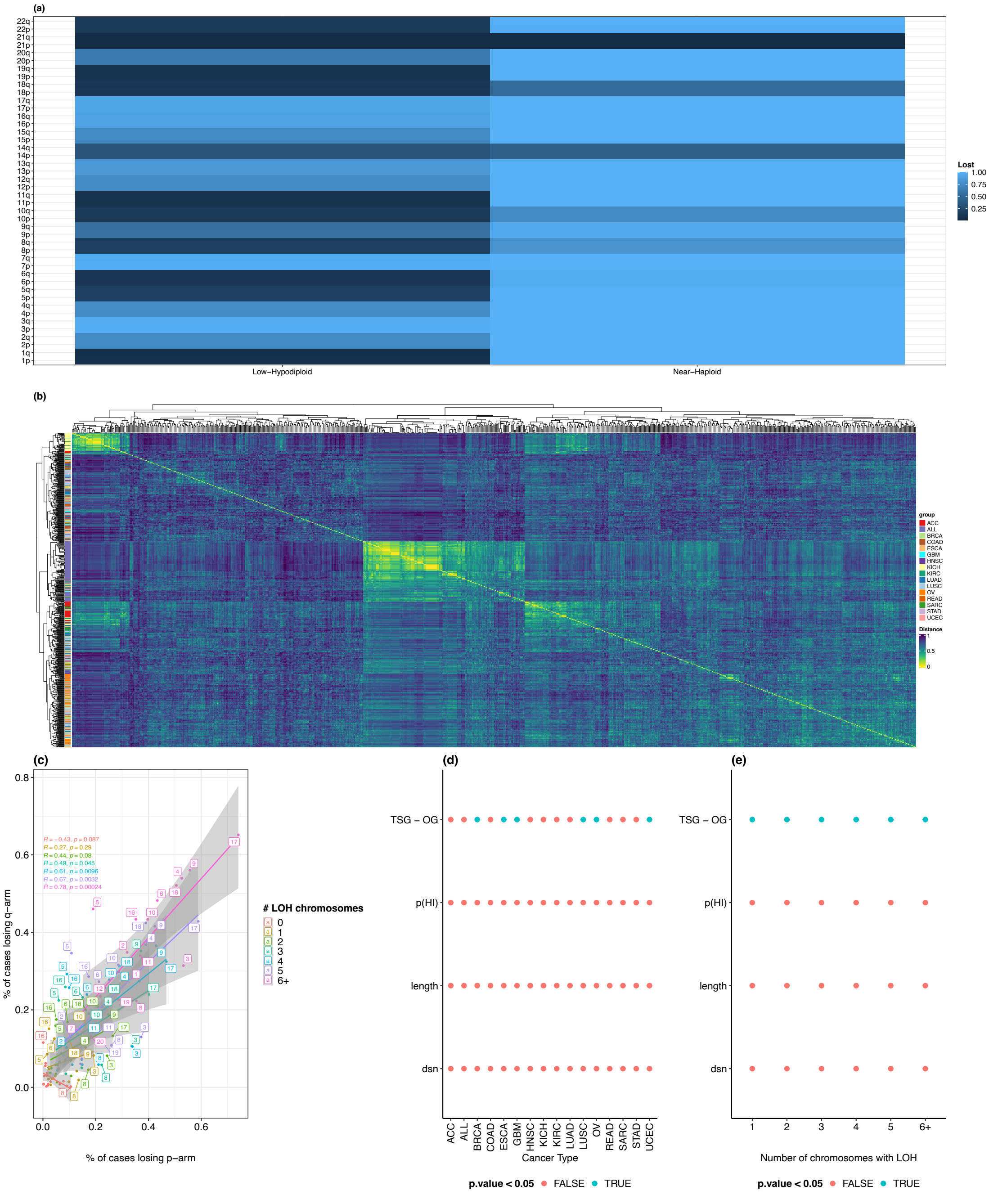

Fig. S3: Related to Fig. 3. Patterns of chromosome loss in hypodiploid tumours. A, Chromosome arm loss rates in near-haploid and low-hypodiploid ALL from the Mitelman database; colour indicates the proportion of cases in which a given chromosome is lost. **B,** Clustering of hypodiploid tumours based on chromosome arm loss patterns. Distance was computed using the Jaccard index between every pair of samples to quantify similarity in the identity of lost vs retained chromosomes. The colour column indicates cancer type. **C,** Correlation between loss rates of p and q chromosome arms within aneuploid tumours in the TCGA dataset, separated by the number of whole chromosomes with LOH. '6+' represents hypodiploid cases. **D,** Tissue-specific generalised linear regression of loss rate in low-hypodiploids on chromosome features including length in Mb, nuclear location (distance from the nuclear surface) from Girelli et al. (2020) [**?**], TSG - OG density score from Davoli et al., and dosage sensitivity, measured as the sum of probabilities of haploinsufficiency (pHI) from Collins et al. (2022) for each gene on the chromosome. **E,** Generalised linear regression of loss rate on chromosome features, split by number of chromosomes with LOH (1, 2, 3, 4, 5 and 6+). ALL was excluded from (E) due to the lack of LOH information in the Mitelman database. (D) and (E) are based on the 20 chromosomes for which we had data from all four predictors (chromosomes 1 to 8 and 10 to 21).

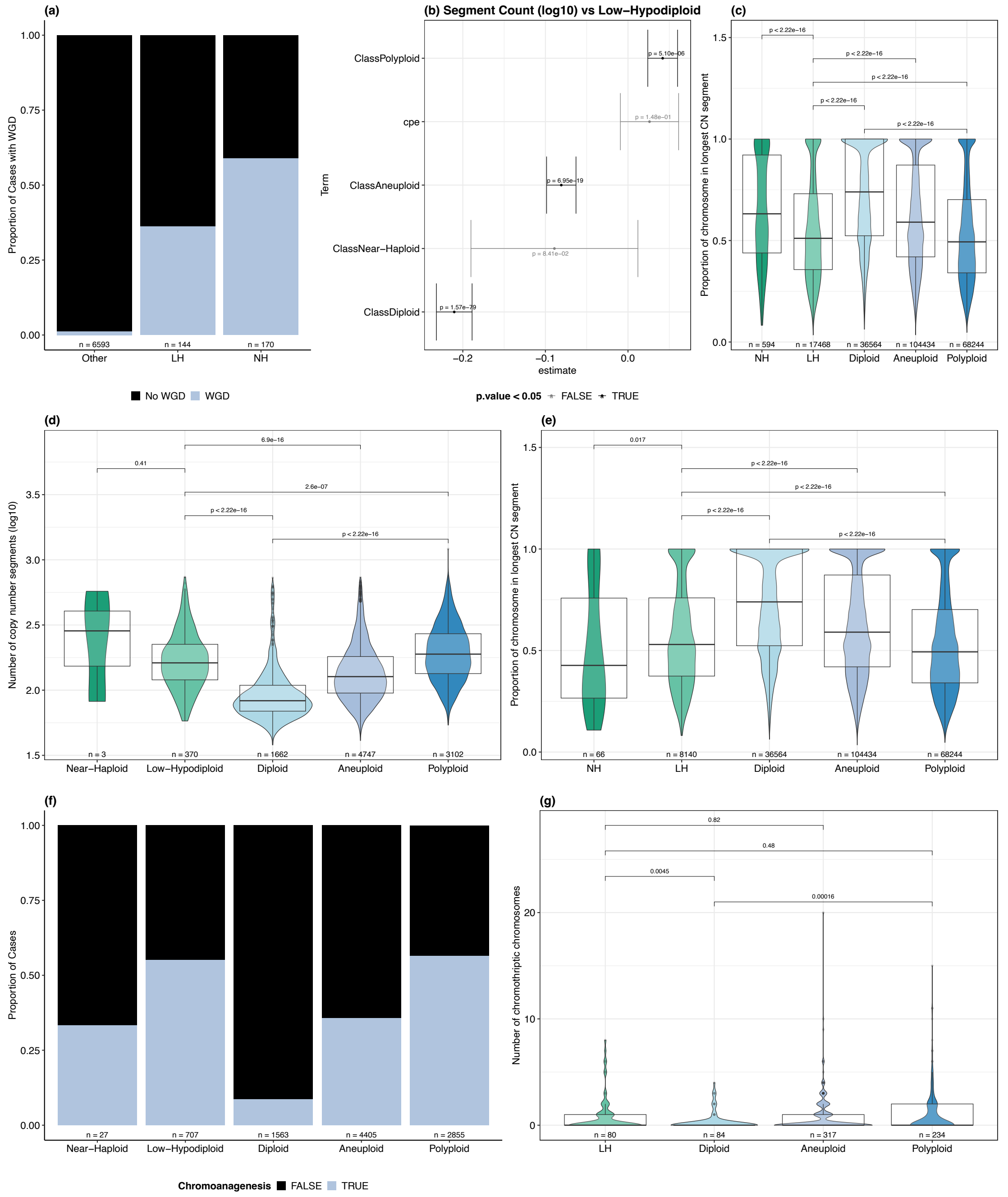

Fig. S4: Related to Fig. 4. Hypodiploid tumours are distinguished by chromosomal instability at multiple scales. A, WGD rate by hypodiploidy level in the Mitelman ALL dataset, but clones with MH score > 0 and autosome count outside the hypodiploid range were reassigned to the 'Other' group and not called as WGD unless they met the ploidy criterion. Multi-clone cases were assigned their lowest ploidy class. **B,** Linear regression of log10-transformed segment count on ploidy class, controlling for purity (cpe, consensus purity estimate from Aran et al. (2015)) and cancer type (not shown), with low-hypodiploidy as the reference level. **C,** Proportion of chromosome in its longest contiguous segment by class (sample size calculated per chromosome per sample). **D,** Number of copy number segments (log10-transformed) by class, excluding doubled hypodiploids. **E,** Proportion of chromosome in its longest contiguous segment by class, excluding doubled hypodiploids. **E,** Proportions of TCGA cases assigned as exhibiting chromoanagenesis by Rasnic & Linial (2021) by ploidy class. **F,** Number of high- or linked to high-confidence chromothriptic chromosomes per sample, from TCGA cases analysed for the PCAWG project by Cortes-Ciriano et al. (2020). Exact p-values for tests with $p < 2.2\text{e-}16$ are reported in Additional File 2

.

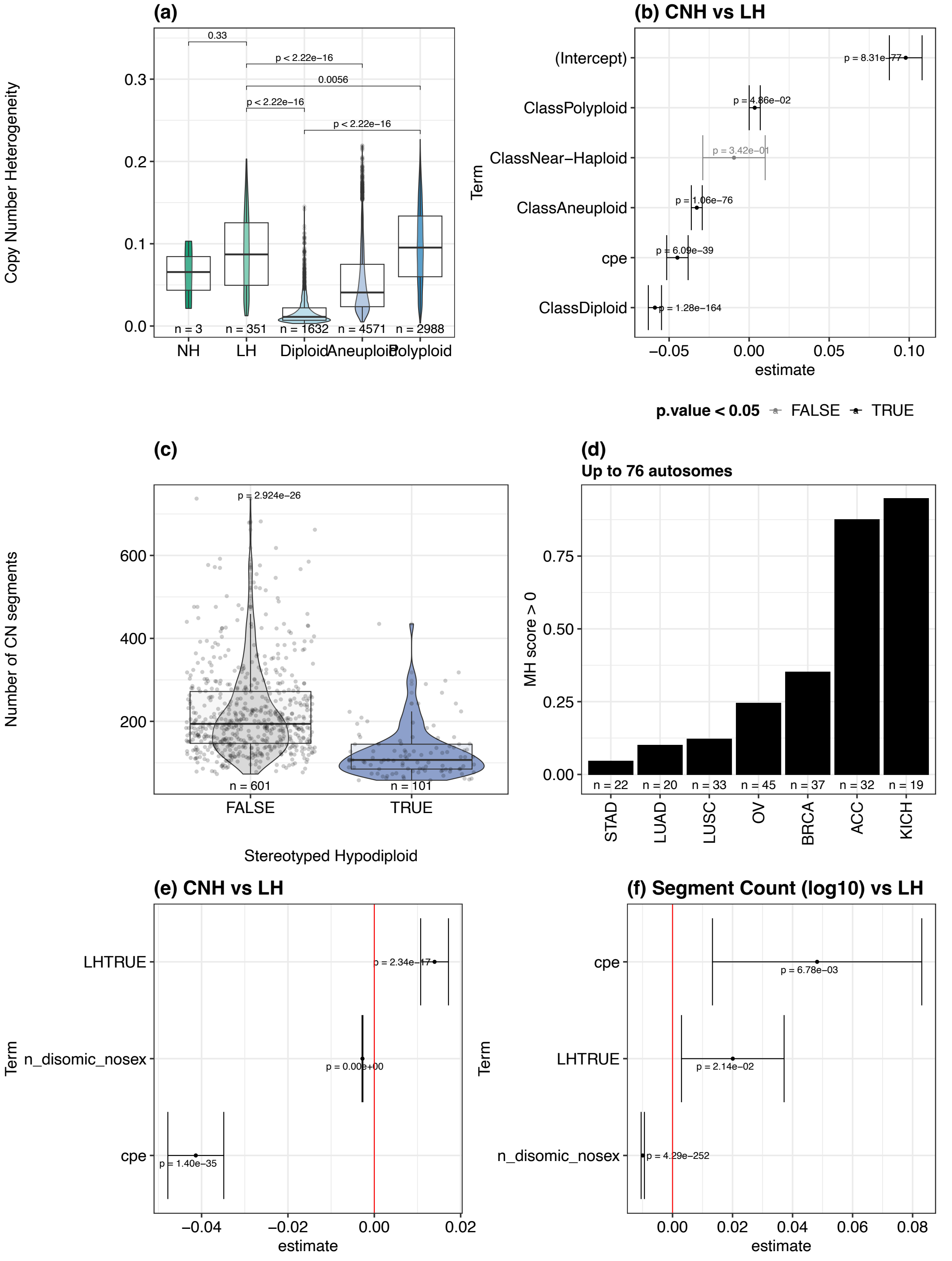

Fig. S5: Related to Fig. 4. Hypodiploid tumours are distinguished by chromosomal instability at multiple scales. A, Distribution of copy number heterogeneity by ploidy class, excluding genome-doubled hypodiploids. **B,** Linear regression of CNH on ploidy class, controlling for purity (cpe) and cancer type (not shown), with low-hypodiploidy as the reference level. **C,** Distribution of segment counts in stereotyped vs non-stereotyped low-hypodiploid tumours (KICH and ACC vs BRCA, LUSC, OV, STAD, LUAD, ESCA, COAD, HNSC, KIRC, SARC, UCEC, READ, GBM). **D,** Sensitivity of the MH score heuristic (tetrasomies - trisomies > 0) in classifying TCGA samples with a hypodiploid history, a positive WGD call and ≤ 76 autosomes as masked hypodiploids. Only cancer types with ≥ 15 doubled hypodiploids with ≤ 76 chromosomes are included. **E,** Linear regression of copy number heterogeneity (CNH) on low-hypodiploid status (against all other samples), controlling for cancer type, tumour purity (cpe) and a measure of aneuploidy (number of disomic autosomes, n_disomic_nosex). **F,** Linear regression of log10(segment count) on low-hypodiploid status (against all other samples), controlling for cancer type, tumour purity (cpe) and a measure of aneuploidy (number of disomic autosomes, n_disomic_nosex). Exact p-values for tests with p <2.2e-16 are reported in Additional File 2.

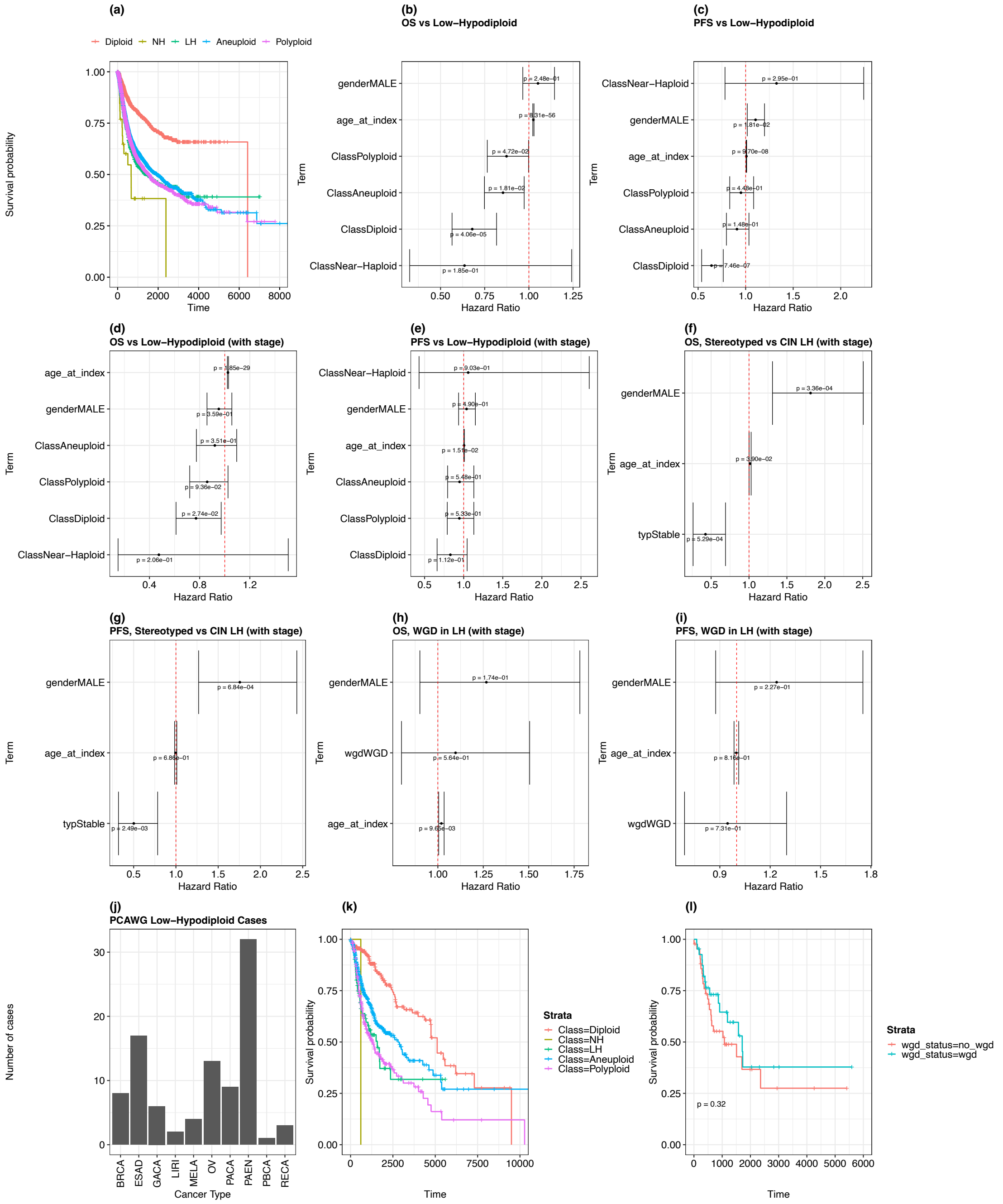

Fig. S6: Related to Fig. 4. Survival analyses. A, Kaplan-Meier curves for progression-free survival (PFS) of TCGA patients by ploidy class **B-E,** Cox proportional hazards regression of survival (OS or PFS as indicated) on ploidy class, controlling for age, sex, race (not shown), cancer type (not shown) and (where indicated) tumour stage. **F-G,** Cox proportional hazards regression of survival on stability within low-hypodiploid tumours, comparing patients with stable (ACC, KICH) vs unstable low-hypodiploid tumours (see Fig. S5), controlling for age, sex, race (not shown) and tumour stage (not shown). **H-I,** Cox proportional hazards regression of survival on genome doubling within low-hypodiploid tumours, controlling for age, sex, race (not shown), cancer type (not shown) and tumour stage (not shown). **J,** Distribution of low-hypodiploid cases by cancer type in the PCAWG dataset, excluding TCGA cases. **K,** Kaplan-Meier curves for overall survival by ploidy class in the PCAWG dataset. **J,** Kaplan-Meier curves for overall survival of PCAWG patients with genome-doubled tumours by WGD status, based on PCAWG WGD calls.

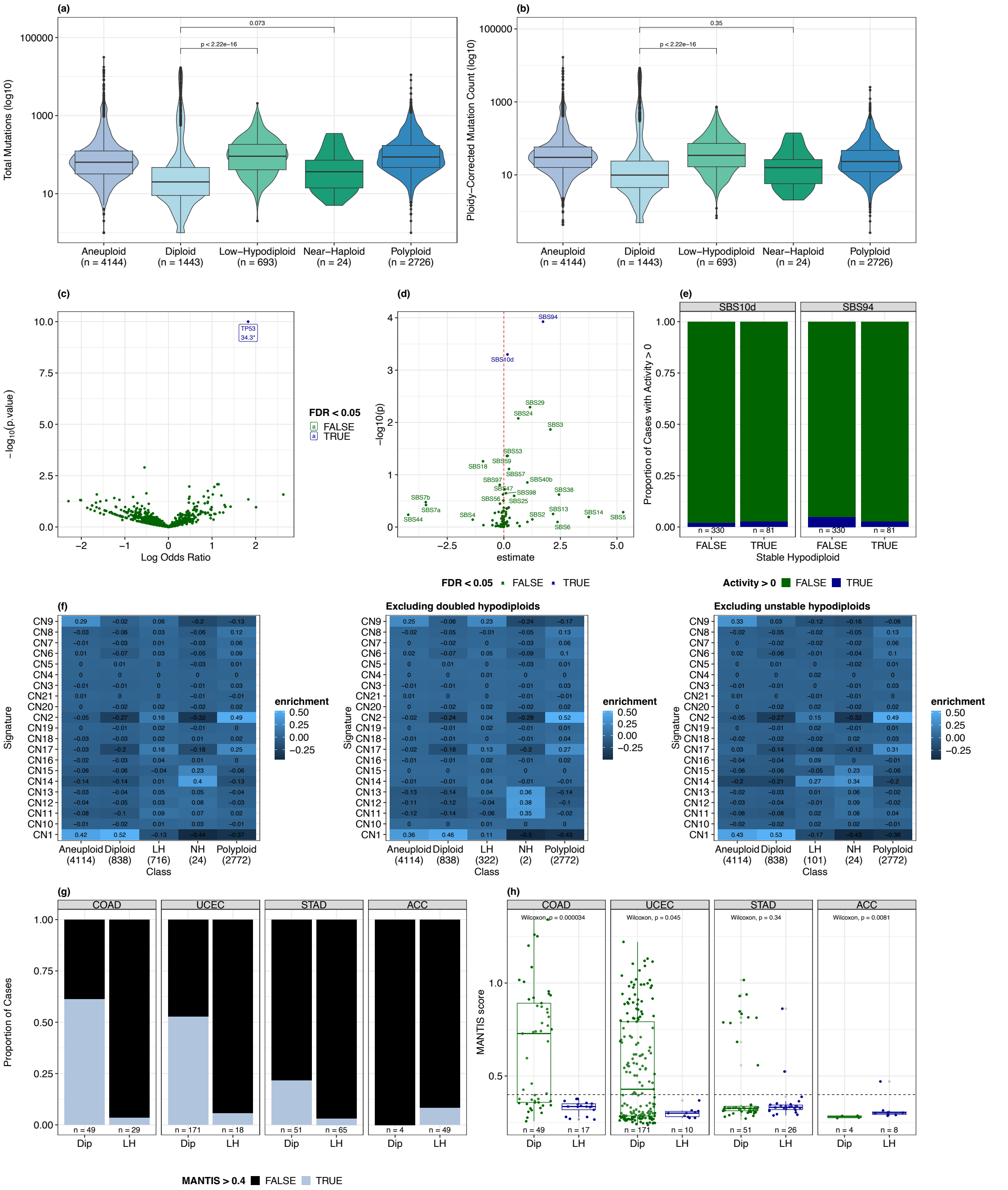

Fig. S7: Related to Fig. 5. Genomic correlates of low-hypodiploidy. A, Total mutation counts by ploidy class. **B,** Mutation rates by ploidy class, mutation counts divided by ploidy. **C,** Genes enriched or depleted for mutations in low-hypodiploid vs less-extreme aneuploid tumours, based on logistic regression controlling for total non-synonymous mutation count and cancer type. -$\log_{10}$(p-values) for genes significant after Benjamini-Hochberg correction are indicated below gene name labels. TP53's -$\log_{10}$(p-value) has been capped at 10 for visibility. **D,** Regression of COSMIC mutational signature activity on ploidy class (low-hypodiploid vs diploid), controlling for patient age, cancer type, tumour purity and total mutation count. **E,** Proportion of low-hypodiploid samples with non-zero SBS10d and SBS94 activity in stereotyped vs non-stereotyped hypodiploid cancer types. **F,** Enrichment of copy number signatures from Steele et al. (2022) in each ploidy class, (left) all samples, (centre) excluding genome-doubled low-hypodiploid and near-haploid cases, (right) excluding low-hypodiploids from non-stereotyped cancer types. **G,** Proportion of cases with MSI-H status (microsatellite instability - high), defined by a MANTIS score > 0.4, in colon (COAD), endometrial (UCEC), stomach (STAD) and adrenocortical (ACC) cancers. Dip, diploid; LH, low-hypodiploid. **H,** Distribution of MANTIS scores excluding genome-doubled hypodiploid tumours. Points are jittered horizontally for visibility. Exact p-values for tests with p <2.2e-16 are reported in Additional File 2.

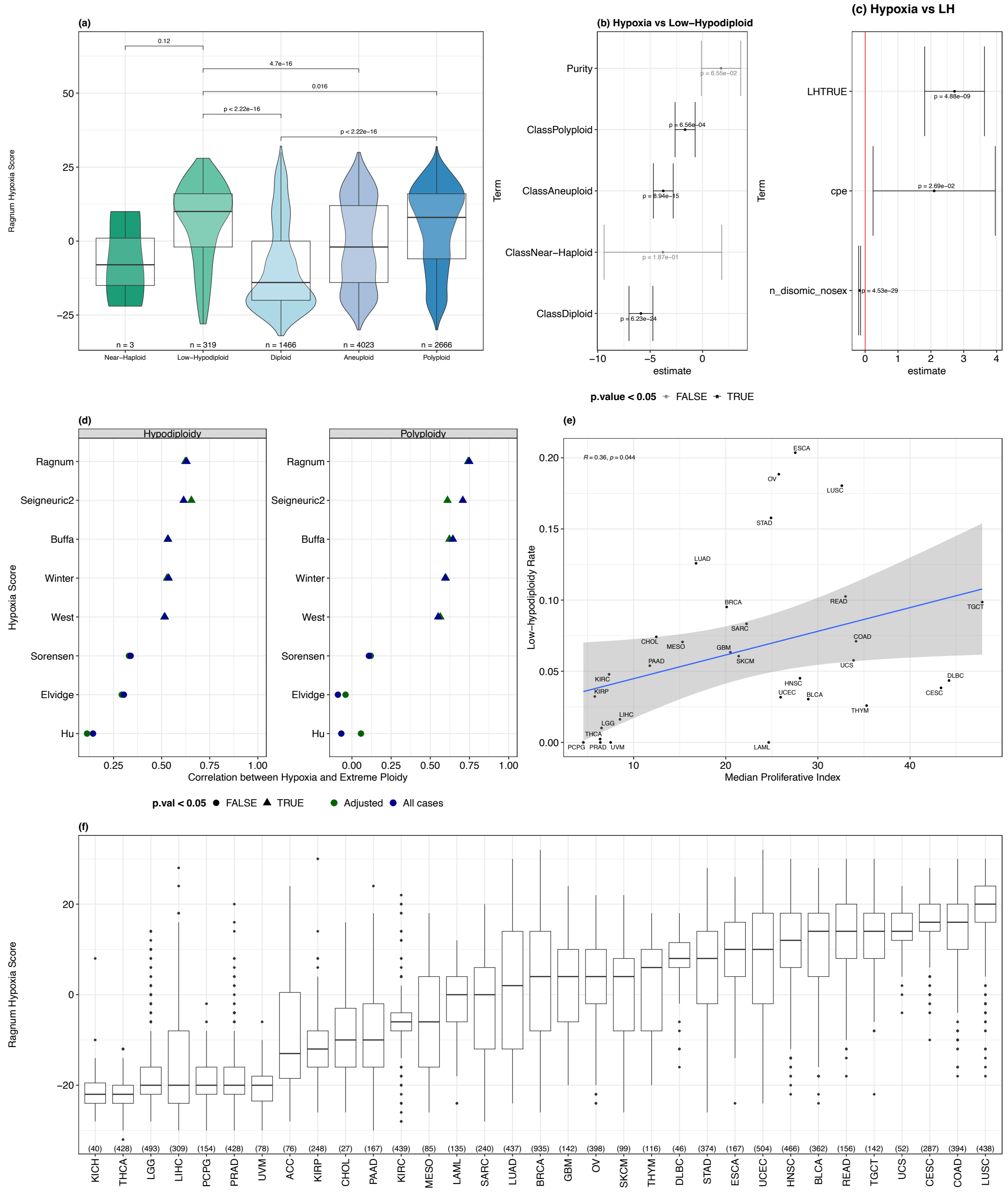

Fig. S8: Related to Fig. 5. Low-hypodiploidy is strongly correlated with hypoxia across cancer types. A, Ragnum hypoxia score by ploidy class after removing genome-doubled hypodiploid tumours. **B,** Linear regression of Ragnum hypoxia score on ploidy class, controlling for cancer type and tumour purity, with low-hypodiploidy as the reference level. **C,** Linear regression of Ragnum hypoxia score on low-hypodiploid status (against all other samples), controlling for cancer type, tumour purity (cpe) and a measure of aneuploidy (number of disomic autosomes, n_disomic_nosex).**D,** Correlations between median hypoxia score and rate of hypodiploidy or polyploidy based on eight different hypoxia signatures. Adjusted points indicate correlations based on median hypoxia scores calculated using non-low-hypodiploid/non-polyploid cases respectively. Distribution of Ragnum hypoxia scores by cancer type, including all ploidy classes. **E,** Cross-tissue correlation between low-hypodiploidy rate and median proliferative index. ACC and KICH were excluded due to their outlying hypodiploidy rates. Proliferative index was calculated based on the median CPM-normalised expression of genes in the metaPCNA proliferation signature from Venet et al. (2011). **F,** Distribution of Ragnum hypoxia scores by cancer type, including all ploidy classes. Exact p-values for tests with p <2.2e-16 are reported in Additional File 2.
