# Supplementary material for: Pervasive chromosomal instability drives the karyotypic evolution of hypodiploid tumours
Source: Genome Med. 2026 May 22;18:70. doi: 10.1186/s13073-026-01632-y (PMC13195901; doi:10.1186/s13073-026-01632-y)
Supplement: Supplementary file 2 — Additional File 2: Supplementary Tables 1-10 [file 13073_2026_1632_MOESM2_ESM.pdf]

Pervasive Chromosomal Instability Drives the Karyotypic Evolution of Hypodiploid Tumours: Supplementary Tables

Comparison	P_Value
Diploid vs Polyploid	0.00e+00
Low−Hypodiploid vs Diploid	2.84e−184
Low−Hypodiploid vs Polyploid	1.00e−03
Near−Haploid vs Low−Hypodiploid	9.00e−03
Low−Hypodiploid vs Aneuploid	4.80e−53

Table S1: Related to Fig. 4c. P-values for Wilcoxon tests comparing (log10) number of copy number segments between ploidy classes.

Comparison	P_Value
Diploid vs Polyploid	0.00e+00
LH vs Diploid	0.00e+00
LH vs Polyploid	1.61e−18
LH vs Aneuploid	1.35e−260
NH vs LH	3.23e−20

Table S2: Related to Fig. S4c. P-values for Wilcoxon tests comparing proportion of chromosome covered by longest contiguous copy number segment between ploidy classes.

Comparison	P_Value
Diploid vs Polyploid	0.00e+00
Low−Hypodiploid vs Diploid	1.18e−88
Low−Hypodiploid vs Polyploid	2.55e−07
Near−Haploid vs Low−Hypodiploid	4.11e−01
Low−Hypodiploid vs Aneuploid	6.87e−16

Table S3: Related to Fig. S4d. P-values for Wilcoxon tests comparing (log10) number of copy number segments between ploidy classes (excluding genome-doubled hypodiploids).

Comparison	P_Value
Diploid vs Polyploid	0.00e+00
LH vs Diploid	0.00e+00
LH vs Polyploid	4.68e−40
LH vs Aneuploid	2.06e−69
NH vs LH	1.60e−02

Table S4: Related to Fig. S4e. P-values for Wilcoxon tests comparing proportion of chromosome covered by longest contiguous copy number segment between ploidy classes (excluding genome-doubled hypodiploids).

Comparison	P_Value
Diploid vs Polyploid	0.00e+00
LH vs Diploid	1.05e−275
LH vs Polyploid	1.47e−01
LH vs Aneuploid	1.30e−110
NH vs LH	2.21e−04

Table S5: Related to Fig. 4e. P-values for Wilcoxon tests comparing copy number heterogeneity between ploidy classes.

Comparison	P_Value
Diploid vs Polyploid	0.00e+00
LH vs Diploid	2.19e−147
LH vs Polyploid	6.00e−03
LH vs Aneuploid	9.72e−44
NH vs LH	3.33e−01

Table S6: Related to Fig. S5a. P-values for Wilcoxon tests comparing copy number heterogeneity between ploidy classes (excluding genome-doubled hypodiploids).

Comparison	P_Value
Diploid vs Polyploid	1.51e−157
Diploid vs Low−Hypodiploid	7.35e−93
Low−Hypodiploid vs Polyploid	8.00e−03
Low−Hypodiploid vs Near−Haploid	1.00e−03
Aneuploid vs Low−Hypodiploid	2.11e−26

Table S7: Related to Fig. 5e. P-values for Wilcoxon tests comparing hypoxia scores between ploidy classes.

Comparison	P_Value
Diploid vs Low−Hypodiploid	7.38e−88
Diploid vs Near−Haploid	7.30e−02

Table S8: Related to Fig. S7a. P-values for Wilcoxon tests comparing total mutation count between ploidy classes.

Comparison	P_Value
Diploid vs Low−Hypodiploid	7.01e−66
Diploid vs Near−Haploid	3.46e−01

Table S9: Related to Fig. S7b. P-values for Wilcoxon tests comparing mutation rate (total mutation count divided by ploidy) between ploidy classes.

Comparison	P_Value
Diploid vs Polyploid	1.51e−157
Diploid vs Low−Hypodiploid	5.30e−56
Low−Hypodiploid vs Polyploid	1.60e−02
Low−Hypodiploid vs Near−Haploid	1.17e−01
Aneuploid vs Low−Hypodiploid	4.69e−16

Table S10: Related to Fig. S8a. P-values for Wilcoxon tests comparing hypoxia scores between ploidy classes (excluding genome-doubled hypodiploids).
